# Supplementary material for: A novel RP-HPLC method for quantification of cholinesterase activity in human blood: An application for assessing organophosphate and carbamate insecticide exposure
Source: PLoS One. 2022 Dec 30;17(12):e0279287. doi: 10.1371/journal.pone.0279287 (PMC9803206; doi:10.1371/journal.pone.0279287)
Supplement: Attachment — Submitted filename: Responces to Reviewer.docx [file pone.0279287.s002.docx]

**Responses to Reviewers**

**Editor and Reviewer Comments:**

According to the Editor’s suggestion, the author followed the PLOS ONE journal authors’ guidelines and revised the whole manuscript as per guidelines.

The reviewer’s comments are in **blue color** and the author’s responses are in **black colo**r. The author incorporated some changes in the revised manuscript and highlighted them in yellow color.

**Response to Reviewer #1**

Thank you very much for considering this manuscript. The author is grateful for the suggestions and recommendations made by you to improve the quality of our manuscript. The manuscript is now revised carefully according to the Editor and reviewer’s suggestions and included it in the detailed point-to-point answers mentioned below.

**Comment 1: The name Chowdhary is misspelled on pages 68, 81, 85, and 88**.

**Response to Reviewer #1** **comment 1:** We thank the reviewer for the correction, we have corrected the error in citation representation and included it in the revised manuscript as per the suggestion. According to your suggestion, We corrected the spelling of Chowdary in Line no. 385, page No. 19 ; Line no. 442, page no. 22, and citation no. 34. According to your suggestion

**Comment 2: Page 51 PDF-based detection should be PDA-based detection**

**Response to Reviewer #****1 comment 2:** We thank the reviewer for the correction, we have corrected the error and included it in the revised manuscript as per the suggestion. We have corrected the error Line No. 79, page No. 4 in the revised manuscript.

**Comment 3: Page 53 section 2.3.1. Isocratic elution was with water-acetonitrile 55:45. However, the last sentence in section 2.3.1 states “All measurements were carried out at room temperature in phosphate buffer (pH-6.88) and acetonitrile”. Please clarify this inconsistency. Did you elute with water-acetonitrile or buffer-acetonitrile? If the eluant contained buffer, please indicate the concentration of phosphate buffer. This is a concern because phosphate buffer may precipitate out of solution in the presence of acetonitrile.**

**Response to Reviewer #1 comment 3:** We apologize for the inconsistency in the data representation; we have corrected and rewritten the **Section “liquid chromatography conditions”** to eliminate the errors, and it has been included in the revised manuscript. All substances were separated chromatographically using a liquid chromatography with a C18 reversed-phase column (150 x 4.6 mm ID, 4.5 µ particle size). The Shimadzu auto-sampler was used to inject samples (20 µL) into the device. Then a 20µL reaction mixture was injected into the RP-HPLC. All measurements were carried out at room temperature. The isocratic composition of mobile phase water–acetonitrile (55: 45, v/v) pumped at a flow rate of 1 mL/min provided the best sensitivity and separation of naphthol compounds. All the standards of 1-naphthyl acetate were prepared in acetonitrile. The temperature in the column oven was kept constant at 25 °C. At a wavelength of 280 nm, the absorbance was measured. and the data were integrated into the software. Generally, the enzyme reaction takes place at pH 7 in the buffer solution. Therefore, the phosphate buffer was used to carry out the reaction in blood samples, and acetonitrile was used to stop the reaction to optimize the kinetics of the reaction at different time periods. Phosphate buffer (pH 6.88) medium was used to carry out the reaction in blood samples and acetonitrile was used for stopped the reaction in blood samples. Only 20 µL Phosphate buffer was used which is very less volume there is no question of precipitation in the column or elution and after adding the acetonitrile to the reaction mixture the phosphate compound present in the buffer precipitated in the reaction mixture after that it was centrifuge and supernatant was collect and filtered with 0.2-micron syringe filter so filtrate is free from cellular debris. As per your valuable suggestion, we incorporated it in the revised manuscript with lines no: 130-142, page no:6-7.

**Comment 4: Page 55 section 2.5. “a solution of 50-µmole of 1-napthol acetate (100-µL)” I think you mean 100 µL of a 50 µM solution of 1-naphthyl acetate? 50 µmole in 100 µL calculates to 0.5 moles/liter, a concentration much too high for your assay.**

**Response to Reviewer #1 comment 4:** We thank the reviewer for the correction; we have corrected the error in the revised manuscript. To confirm that AChE is involved in the conversion of 1-naphthol acetate to 1-naphthol, the following assay was performed in 50 µL clean water (LC-MS grade) add 50 µL solution of 50 µM of 1-naphthol acetate was added and followed by AChE (2 unit/µL) in 400µL phosphate buffer (pH 6.88). After different time intervals, the reaction was stopped using with 500 µL acetonitrile and filtered using a 0.2µ syringe filter and a 20µL reaction mixture was injected into RP-HPLC. So, the concentration of 1-naphthol acetate was not too high. As per your valuable suggestion, we incorporated it in the revised manuscript with lines no: 187-192, page no:9.

**Comment 5: Page 55 section 2.5. “AChE (2 unit/µL)” implies that you used a commercial preparation of AChE whose activity was defined by the supplier. If this guess is correct, please add the name of the supplier, the catalogue number and the AChE species (human, horse, electric eel?)**

**Response to Reviewer #1 comment 5:** AChE (Human) was procured from Sigma-Aldrich with CAS-No: 9000-81-1 and product number of C0663. The same was used for the reaction in our manuscript. As per your valuable suggestion, we incorporated it in the revised manuscript with lines no: 105-106, page no:5.

**Comment 6: Before you describe assays that use blood, you need to describe what you mean by blood. It is important for the reader to know whether you used whole blood (never frozen) or whole blood that was frozen and thawed, or isolated red blood cells. A separate section describing blood should be included before section 2.3.
Response to Reviewer #1 comment 6:** The 2 ml whole blood was collected from healthy volunteers and then subjected to a reaction without freezing the samples. A sample volume of 10 µL from the 2 ml collected blood was used for the reaction. The blood samples were collected from venous blood and placed in test tubes. Capillaries and test tubes should be heparinized (to prevent blood clotting) and dried (to prevent uncontrolled sample dilution). To prevent contamination of the samples by OPs and carbamates during collection, the skin must be cleaned before sampling. Similarly, The RBC were separated from whole blood by centrifuging it at 500 x g for 10 min at 4 degrees C. Aspirate the supernatant (plasma) and add cell wash buffer to the erythrocyte pellet. Discard the supernatant and repeat washing with 2 ml of PBS (Phosphate buffered saline) in order to remove the residual plasma and any residual OPs or carbamates that may be present in the blood [36]. As per your valuable suggestion, we incorporated it in the revised manuscript with lines no: 114-124, page no:6.

**Comment 7: Section 2.5 is entitled “Assay optimization in water”. This title is confusing because the assay contains 400 µL phosphate buffer plus 100 µL of naphthyl acetate in acetonitrile plus 50 µL water. Please clarify why 50 µL water optimizes the assay.**

**Response to Reviewer #1 comment 7:** To confirm that AChE is involved in the conversion of 1-naphthol acetate to 1-naphthol, the following assay was performed. 50 µL clean water (LC-MS grade) add with 50 µL solution of 50 µM of 1-naphthol acetate (100 µL) was added and followed by AChE (2 unit/µL) in 400µL phosphate buffer (pH 6.88). After different time intervals, the reaction was stopped using with 500 µL Acetonitrile and filtered using a 0.2µ syringe filter and collect the filtrate of 20µL reaction mixture was injected in RP-HPLC under the same conditions mentioned above. As per your valuable suggestion, we incorporated it in the revised manuscript with lines no: 186-192, page no:9

**Comment 8: Page 56 and page 57 “Finally, 10 µL of blood sample was standardized for the estimation. Subsequently, 10 µL of 50 µM 1-naphthol acetate was added to the blood followed by 280 µL of phosphate buffer.” This order of addition is a problem. If you added 10 µL of 50 µM naphthyl acetate dissolved in acetonitrile to 10 µL blood, the acetonitrile would inactivate the activity of AChE. A more likely order of addition is 10 µL blood added to 280 µL buffer, followed by 10 µL of 50 µM 1-naphthyl acetate.**

**Response to Reviewer #1 comment 8:** We thank the reviewer for the comment, we have corrected the error and also included a schematic figure representing the process in the revised manuscript. Firstly 10µL blood was added in 280 µL phosphate buffer was taken and, thereafter 10µL of 50µM 1- naphthol acetate was added and incubated up to 20 minutes, next add 700 µL acetonitrile for stopped the reaction and filtered. 20 µL filtrate was injected to RP-HPLC system. As per your valuable suggestion, we incorporated it in the revised manuscript with lines no: 193-210, page no:10

This procedure is depicted in the schematic figure below.

**Comment 9: Page 56. “The complete conversion of 1-naphthol acetate to 1-naphthol was achieved using 50 µmole concentration in water”. According to page 52 section 2.2, 50 µM 1-naphthyl acetate was in acetonitrile, but on page 56 you say the naphthyl acetate was in water. Was your naphthyl acetate solution in acetonitrile or in water? Is naphthyl acetate soluble in water at 50 µM?**

**Response to Reviewer #1 comment 9:**1-naphthol acetate solution was prepared in acetonitrile, not in water. We apologize for the error in the representation; we have used acetonitrile as the solvent, and the same has been corrected in the revised manuscript. As per your valuable suggestion, we incorporated it in the revised manuscript with lines no:135- 136, pages no:6 -7.

**Comment 10: Page 56. “Similarly, in 1 mL phosphate buffer run on HPLC.” I do not understand this sentence.**

**Response to Reviewer #1 comment 10:** Similarly, in 20 µL of phosphate buffer was injected into the system and run-on RP-HPLC, where no peak was observed, which acts as a control. The same has been corrected in the revised manuscript. 1ml phosphate buffer was not run in RP-HPLC. As per your valuable suggestion, we incorporated it in the revised manuscript with lines no:207-210, page number 10;

**Comment 11: Page 56. The description of the method for preparing red blood cells should be in a separate section, before section 2.3.**

**Response to Reviewer #1 comment 11:** We have separated the red blood cells preparation part as per the suggestions of the reviewer. The separation method of RBC is mentioned in 2.2.1 section “**Collection of blood and isolation of RBC”**

The 2 ml whole blood was collected from healthy volunteers and then subjected to a reaction without freezing the samples. A sample volume of 10 µL from the 2 ml collected blood was used for the reaction. The blood samples were collected from venous blood and placed in test tubes. Capillaries and test tubes should be heparinized (to prevent blood clotting) and dried (to prevent uncontrolled sample dilution). To prevent contamination of the samples by OPs and carbamates during collection, the skin must be cleaned before sampling. Similarly, The RBC were separated from whole blood by centrifuging it at 500 x g for 10 min at 4 degrees C. Aspirate the supernatant (plasma) and add cell wash buffer to the erythrocyte pellet. Discard the supernatant and repeat washing with 2 ml of PBS (Phosphate buffered saline) in order to remove the residual plasma and any residual OPs or carbamates that may be present in the blood. As per your valuable suggestion, we incorporated it in the revised manuscript with lines no: 114-124, page no:6.

**Comment 12: Page 57 “All the blood samples collected from healthy volunteers, farmers and intentionally ingested people were extracted”. Please explain what you mean when you say blood samples were extracted. The word extraction means you removed something from the blood. My understanding of the text is that you used whole blood diluted into buffer. I do not see an extraction step.**

**Response to Reviewer #1 comment 12:** We have corrected the phrase with subjected to “extraction” in the revised manuscript as per the comment from the reviewer. All the blood samples were collected from healthy volunteers, and farmers exposed to the pesticide while spraying, and suicidal patients who ingested pesticides while hospitalized. All blood samples were subjected to the reaction procedure and analysed on the developed method and AChE levels were estimated and mentioned in Fig 1. The blood samples were subjected to reaction and then the conversion was monitored on the HPLC system which we have represented as an extraction. After completion of the reaction for 20 minutes, added with acetonitrile was used to stop the reaction to inhibit AChE enzyme activity on RBC, and thereafter the reaction mixture was filtered using 0.2µ syringe filters for filtration and collect the filtrate and discard the other cellular debris. The 20µL filtrate was injected into the RP HPLC system, which we may have represented as an extraction, where actually it is not an extraction. It is only filtration to remove blood cell-related impurities. We incorporated it in the revised manuscript lines no: 221-232 page no: 11

**Comment 13: Table 1 title. Suggested title:** **Literature review of HPLC methods for assaying AChE activity and inhibition.**

**Response to Reviewer #1 comment 13:** We have modified the title as per the suggestion of the reviewer and included in the revised manuscript. As per your valuable suggestion, we incorporated it in the revised manuscript with lines no: 241, page no:11.

**Comment 14: Sections 3.1.3, 3.1.4, 3.1.5, 3.1.6 and 3.1.7. Were these values measured in the presence of blood or in the absence of blood? Please clarify this point. I am guessing that no blood or AChE was present in the results for sections 3.1.3 to 3.1.7. Please add a statement that the assays did not include** **AChE or blood.**

**Response to Reviewer #1 comment 14:** We thank the reviewer for the comment. During the method validations AChE or blood were not used. and was incorporated into sections 3.1.3 to 3.1.7 as per reviewer’s suggestions. As per your valuable suggestion, we incorporated it in the revised manuscript with lines no: 145 page no: 7

**Comment 15: Figure 4 and 5 legends. I think Figures 4 and 5 represent AChE activity, but I am not certain. If they do represent AChE activity, please modify the figure legends as follows: “****1-naphthol acetate conversion to 1-naphthol by AChE in blood at different time intervals.”**

**Response to Reviewer #1 comment 15:** As per the reviewer’s suggestion we have modified the figure legends in the revised manuscript. figure 4 becomes figure 5; we incorporated it in the revised manuscript with lines no: 311 page no:16

- Fig 5. 1-naphthol acetate conversion to 1-naphthol by AChE in blood at different time intervals

**Comment 16: Figure 5 legend “****Concentration of 1-naphthol formed by AChE in blood at different time intervals.”**

**Response to Reviewer #1 comment 16:** As per the reviewer suggestion we have modified the figure legends in the revised manuscript. figure 5 become figure 6; we incorporated it in the revised manuscript with lines no: 312 page no:16

- Fig 6. Concentration of 1-naphthol formed by AChE in blood at different time intervals

**Comment 17: Tables 4 and 5. Please define units/ml. Is it µmoles naphthyl acetate hydrolysed per ml?**

**Response to Reviewer #1 comment 17:** The 1-naphthol acetate was hydrolysed per ml but the enzyme activity was expressed in units/ml. We cannot express the enzyme activity in only ml. In Tables 4 and 5, the enzyme activity was expressed in units/ml. Therefore, it was mentioned.

**Comment 18: Tables 4 and 5. Which data in which figures were used to calculate units of activity?**

**Response to Reviewer #1 comment 18:** Table 4 and Table 5 represents the levels and concentrations of AChE in the blood collected from control, Exposed and Intendedly ingested persons which we have not represented in any of the figures in our manuscript. Each batch of analysis was included with a standard 50µM 1-naphthol and 1 naphthol acetate in the analysis. The calculation was represented with the standard concentrations of the particular batch. The concentration of formation of a product that is 1-naphthol was considered for the calculation of enzyme activities which is represented in figure 5.

**Comment 19: Please use the Spell Check feature in Microsoft Word to correct the many errors in the text.**

**Response to Reviewer #1 comment 19:** We thank the reviewer for the suggestion and as per the recommendation we have corrected the errors in the text of the revised manuscript by using Spell Check feature in Microsoft Word.
