# Supplementary material for: A novel RP-HPLC method for quantification of cholinesterase activity in human blood: An application for assessing organophosphate and carbamate insecticide exposure
Source: PLoS One. 2022 Dec 30;17(12):e0279287. doi: 10.1371/journal.pone.0279287 (PMC9803206; doi:10.1371/journal.pone.0279287)
Supplement: Attachment — Submitted filename: Responces to Reviewer Comments.docx [file pone.0279287.s001.docx]

**Responses to Reviewers**

Major comment: A major fallacy in the present work is the notion that hydrolysis of naphthyl acetate is a property of acetylcholinesterase. The published literature reports that butyrylcholinesterase in human plasma hydrolyzes naphthyl acetate, but that acetylcholinesterase in red blood cells does not. There was no attempt to determine whether the activity is in plasma or in red cells. It is acceptable to devise a new method to assay cholinesterase activity. However, the comments in the manuscript about the Ellman assay, indicate the authors have no experience with the Ellman assay. Criticizing the time-honored Ellman assay does not benefit the present report.

**Response:** We thank the reviewer for their valuable comments and helping our improve the manuscript and helping us understand the critical points involved for the manuscript. We have performed the assay using water as a sample and then adding AChE to understand the hydrolysis of 1-naphthol acetate. We observed that hydrolysis has been occurred and the conversion to 1-naphtol has been identified on the HPLC chromatogram. The same has been performed using buffer solution with and without AChE and confirmed its conversion by 1-naphthol acetate (Page 13, Section 3.2). Also, an in-silico done by Chowdary S et al. in 2018 has reported that 1-napthol acetate is more favourable to Ache over Bche which was observed in our study as well. We apologise the criticism on the Ellman assay, the method has been a gold standard for many years, our only intention was to report that HPLC method is a more confirmatory method than the calorimetric method (Page 20, Paragraph 1)

**Comment 1**: Introduction: The statement that the Ellman assay does not provide speedy results is incorrect. I use the Ellman assay and get results in 40 seconds.

**Response:** We have corrected the statement in the revised method and removed the statement. The Ellman assay is a calorimetric method where as our method is a chromatographic method which is a confirmatory result as compared to calorimetric method.

**Comment 2:** 1-naphthol A should be written 1-naphthol acetate

**Response:** We have abbreviated1-naphthol A to 1-naphthol acetate as per the reviewer’s suggestion.

**Comment 3:** Typing error “Using 1-naphthol acetate as a substrate instead of AChE is expected to be a reliable way to measure cholinesterase activity, avoiding the drawbacks of AChE [34,35]”. In this sentence AChE should be acetylthiocholine.

**Response:** We thank the reviewer for the correction, we have corrected the error and included in the revised manuscript as per the suggestion.

**Comment 4:** typing error? “4.5 m particle size”. Should this be 4.5 µ particle size?

**Response:** The typing error has been corrected in the revised manuscript.

**Comment 5:** Please clarify the meaning of “blood”. Is it whole blood or plasma or serum? If whole blood, has it been stored frozen? Red blood cells lyse when whole blood is frozen. The lysed red blood cells release hemoglobin. Did hemoglobin precipitate out of solution when you stopped the reaction with acetonitrile?

**Response:** Thank you for comment. The authors would like to convey the response that clarifies the blood, i.e., The blood samples were collected from venous blood into test tubes. Capillaries and test tubes should be heparinized (to prevent blood clotting) and dried (to prevent uncontrolled sample dilution). To prevent contamination of the samples by OPs and carbamates during collection, the skin must be cleaned before sampling. We also focused on AChE activity in whole blood as well as in erythrocytes, we have used whole blood for the analysis. The whole blood has been lysed upon addition of acetonitrile and the RBCs were filtered through 0.2µ syringe filters and the filtrate was used for HPLC analysis

**Assay optimization in whole blood**

The basic methodology is simple. To check the enzyme activity buffer solutions with 1-naphthol and 1-naphthol acetate were injected separately into the HPLC system and no conversion of substrates was observed. We have also used phosphate buffer with and without ChE to observe the 1-napthol acetate conversion to 1-napthol which clearly indicates that 1-napthol acetate is being converted to 1-napthol by acetylcholinesterase. Further, blood samples from people of our institute who are not exposed to any pesticide sprays were used for observing the reaction and impact of ChE in conversion of the substrate. The peak areas of the substrate during conversion has been represented in the Figure-4. The curves shows the conversion of 1-naphthol acetate to 1-naphthol at different time points. The concentration of 1-naphthol conversion in blood at different time intervals in represented in Figure-5.

Additionally, to prove our method the reaction was also carried out with RBC with 1-napthol acetate. The RBC were separated from whole blood by centrifuging it at 500 x g for 10 min at 4 degrees C. Aspirate the supernatant (plasma) and add cell wash buffer to the erythrocyte pellet. Discard the supernatant and repeat washing with 2 ml of PBS in order to remove the residual plasma and any residual OP or carbamates that may be present in the blood (Reiner, Elsa, (2006). AChE activity was measured either in unwashed erythrocytes or in erythrocytes that had been washed with buffer or saline in order to remove the residual plasma and any residual inhibitors or oximes that may be present in the blood. In this experiment, we collected erythrocytes (whole blood without plasma) kept at room temperature for use in the experiment. After collecting packed erythrocyte samples and adding 1-naphthyl acetate, the reaction mixture was filtered with 0.22 microns, and the filtrate was collected and loaded into the RP-HPLC. The samples were not frozen or stored at a cold or cool temperature, so there was no risk of hemolyses in the sample, the red blood cells (RBC) was not lysed, and haemoglobin did not precipitate with acetonitrile. When AChE activities are measured in erythrocytes separated from plasma by centrifugation, they are measured either in unwashed erythrocytes or in erythrocytes that have been washed with buffer or saline in order to remove the residual plasma and any residual inhibitors or oximes that may be present in the blood. {Reiner, Elsa (2006). Toxicology of Organophosphate & Carbamate Compounds || Methods for Measuring Cholinesterase Activities in Human Blood., 199–208. doi:10.1016/B978-012088523-7/50015-6}. We describe the blood collection and assay procedures that were incorporated into the revised manuscript. The isolation of RBC is little-bit difficult task not easy so our method focused on whole blood selected as a matrices.

**Assay optimization of RBC AChE activity in human blood**

The RBC was separated from whole blood by centrifuging it at 500 x g for 10 min at 4 degrees C. Aspirate the supernatant (plasma) and add cell wash buffer to the erythrocyte pellet. Discard the supernatant and repeat washing with 2 ml of PBS in order to remove the residual plasma and any residual OP or carbamates that may be present in the blood (Reiner, Elsa, (2006).

Erythrocyte samples and the substrate (1-naphthol acetate) were put in a buffer at room temperature for 20 minutes. The reaction was stopped when acetonitrile was added, and the samples were filtered with 0.22-micron syringe filter paper. The filtrate was then put into the RP-HPLC system. The conversion of 1-naphthol acetate to 1-naphthol, catalysed by the AChE present in the RBC membrane, was followed by the simultaneous disappearance of the former and the appearance of the latter on the chromatograms. Several concentrations and blood volumes were tried, and a 50 µM concentration of 1-naphthol acetate was standardised for the reaction and estimation. Finally, 10 µL of blood was standardised for the estimation. Subsequently, 10 µL of 50 µM 1-naphthol acetate was added to the blood, followed by 280 µL of phosphate buffer. The reaction was monitored every 5 minutes. The complete conversion of 1-naphthol acetate to 1-naphthol was achieved using a 50 µM concentration in water within 20 minutes of the reaction initiation, which proves that the enzyme is very reactive and responsible for the conversion of 1-naphthol acetate to 1-naphthol. Similarly, in 20 µL of phosphate buffer was run on RP-HPLC, no peak was observed, which acts as a control.

**Comment 6:** Naphthol acetate is hydrolyzed by butyrylcholinesterase, but not by acetylcholinesterase. P.W. Zapf and C.M. Coghlan (1973) ClinicaChimica Acta 44, 237-242. A kinetic method for the estimation of pseudocholine esterase using naphthyl acetate substrate.
The introduction assumes you measured AChE activity, but this assumption is incorrect.
You measured butyrylcholinesterase activity. In the older literature, butyrylcholinesterase was called pseudocholinesterase. Please modify the text to acknowledge you measured BChE activity.

**Response:** In 50-µl clean water (LC-MS Grade), a solution of 50-µmole of 1-napthol acetate (100-µl) was added and followed by AChE (2 unit/µl) in phosphate buffer (pH-6.88) (400µl). After different time interval the reaction was stopped using 500-µl Acetonitrile and filtered using a 0.2µ syringe filter and a 10µl reaction mixture was injected in HPLC and confirmed that AChE was involved in the conversion.

Also 1-NA can be an attractive chromogenic substrate than the Acetylthiocholine or Butyryl thiocholine for the measurement of AChE activity. 1-NA to be a better alternative substrate for AChE activity than acetylthiocholine (ATCh) in terms of lower Km value. Its specificity appeared at least similar to ATCh. Therefore, the authors proposed that 1-NA can be an attractive chromogenic substrate for the measurement of AChE activity, and it possesses the potential to detect organophosphorus pesticide (OP) poisoning. The same has been observed in the study by Chowdary S et al., 2018

**Also, 1-Naphthol acetate is hydrolyzed by both enzymes, such as AChE in RBC/whole blood while BuChE/PChE hydrolysed in plasma only (**Reiner, Elsa (2006). Toxicology of Organophosphate & Carbamate Compounds || Methods for Measuring Cholinesterase Activities in Human Blood.**), but we were focused only on AchE activity on RBC/whole blood samples and not focused on BuChE in plasma.**

**BuChE activities are measured not only to evaluate inhibition by OPs or CMs but also to diagnose a wide range of physiological or pathological conditions reflected in BuChE activities. These measurements are done in serum or plasma and not in whole blood. Therefore, more data on BuChE than AChE activities are available, and more methods have been developed for the analysis of plasma or serum than in whole blood (**Reiner, Elsa (2006). Toxicology of Organophosphate & Carbamate Compounds || Methods for Measuring Cholinesterase Activities in Human Blood.**). That’s why we used to focus on AChE activity on isolated RBC from whole blood. Therefore, in this manuscript, we measured only the AChE activity on whole blood but did not focus on the BuChE activity in the plasma; this paper assumes we only focused on AchE activity. We have not measured butyrylcholinesterase activity because we have not used plasma samples, therefore, there we have not considered the activity of butyrylcholine esterase (Page 19).**

**Comment 7:** Section 2.6. Please explain what you mean by “These samples were extracted”

**Response:** All the blood samples collected from Healthy people, Farmers and suicidal patients were used for extraction and analysis on the developed method.

**Comment 8:** Section 2.5. Please describe the filter that was used to prepare samples for HPLC. Was it a paper filter? Did the filter separate all the proteins from the substrate and product? Blood contains many small molecules. How did you separate contaminating small molecules from naphthol acetate and naphthol?

**Response:**0.2µ syringe filters were used for filtration. The filtration was carried out by gently applying pressure on the piston of the syringe and the filtrate was collected into 200 µL inserts placed into the 1.5ml Screwcap vials.

**Comment 9:** Table 1 cites reference 54 for hydrolysis of naphthyl acetate by acetylcholinesterase. I checked reference 54 and found that the substrate was acetylthiocholine and the enzyme was a commercial preparation of AChE. Naphthyl acetate was not used in reference 54.

**Response:** We tried to emphasise on different methods in Table 1, that has been developed previously for the identification and estimation of acetylcholinesterase. The intention of our inclusion with reference 54 was about the method that the authors employed for identifying acetylcholinesterase on HPLC where they used Methanol: Water: Diethyl Amine in the ration of 40:60:0.05 while our method was employed to identify 1-napthy acetate with simpler mobile phase of acetonitrile–water in the ratio of 45:55 at 1ml/min flow rate which is quite solvent saving and easier to employ.

Additionally, we thank you for your valuable suggestion, We would to explain that, as done by *Chowdhary S, Bhattacharyya R, Banerjee D, Biochime 2018,* they observed Erythrocyte acetylcholinesterase (AChE) is a preferred biomarker for the detection of organophosphorus poisoning.The in silico approach was performed to understand the fitness and the Total Interaction Energy (TIE) of substrates for AChE. The alternative substrates for AChE were screened in terms of high Goldscore and favorable TIE in comparison to acetylcholine (ACh)-AChE complex and other relevant esterases. Among the screened substrates, 1-Naphthyl acetate (1-NA) exhibited the most favorable interaction with AChE in terms of highest TIE and corresponding high Goldscore. The Molecular Dynamic (MD) simulation of the 1-NA-AChE complex showed a stable complex formation over a period of 5 ns. The results obtained in the in silico studies were validated in vitro using pure erythrocyte AChE and hemolysate.

1-NA to be a better alternative substrate for AChE activity than acetylthiocholine (ATCh) in terms of lower **Km value**. Its specificity appeared at least similar to ATCh. Therefore, the authors proposed that 1-NA can be an attractive chromogenic substrate for the measurement of AChE activity, and it possesses the potential to detect organophosphorus pesticide (OP) poisoning}”.

**Comment 10:** “However, this technique has their own limitation, as well as several mL of blood is necessary”. This statement is incorrect. Ellman uses 50 µl AChE in a 3 ml assay. I use 10 µl plasma in a 2 ml Ellman assay.

**Response:** We agree with the reviewer comment and apologise for our statement, we have deleted the statement in the revised method. However, these days the most applied technique to determine cholinesterase activity is Ellman’s colorimetry. It is suitable for the assessment of biological samples, for the identification of AChE and the determination of their efficacy. But its limitation lies in strong interaction with hemoglobin during whole blood sample analysis. Furthermore, Ellman’s colorimetry cannot be uncritically used to assay the oxime reactivators. It may not be used in various protocols for the determination of pesticide or nerve agent exposure. This method used only for hemoglobin-rich samples. Johnson and Russel reported radiometric assay is a practical approach for protocols where high sensitivity is needed. Fluorometric methods are useful for sensitive determination within kinetic studies at very low enzyme concentrations. Similarly, Biosensors seem to be suitable for OP and carbamate identification. The AChE activity determination approach needs to fulfill several requirements like sufficiently sensitive, selectivity for AChE activity is necessary. Our method is capable for the assessment of biological samples as well as for kinetic studies and new drug discovery at very low level. Earliest techniques were developed mainly for overall AChE activity determination without accuracy. All techniques used either natural substrate AChE or artificial substrates. Our new reported method extensively improved in respect of sensitivity, selectivity, great precision, and time factor by introducing new biomarker 1-Napthol and 1-napthol acetate as a substrate. Great potential lies in use of our methods, which creates new possibilities in this field.

This method developed on the basis for a variety of tests that might have used to measure biological effect. The principles that developed method here could be used in clinical settings as enzyme activities that are impaired by specific exposures are discovered. Arsenic, for example, is known to interact with a wide variety of sulfur containing proteins. This work will also have an impact on exposure to polycyclic aromatic hydrocarbons (Page 17).

**Comment 11:** “the possibility of reaction between butyl acetylcholine esterase and 1-napthol acetate is minimal, while similar observation was observed earlier [51-53].” References 51-53 do not support your opinion that AChE rather than BChE is responsible for hydrolysis of naphthyl acetate in your assay. It is suggested that you check the specificity of your assay by separating plasma from red blood cells in a blood sample that has never been frozen, and then measure hydrolysis of naphthyl acetate by plasma using your method. Plasma contains BChE. You will find high activity in plasma.

**Response:** We agree with the reviewer comments that plasma has high activity from BChE, however AChE has been more favourable in interaction towards 1-naphthol acetate as compared to BChE. The same has been studied by Chowdary S et al., 2018 and the in-silico interaction with different esterase have revealed the same as reported in our study. We have also modified the references to support our statement and removed other references.

**Comment 12:** “However, Bche has been studied primarily concerning toxicity caused by drug and drug-drug interactions.” This is an outdated idea. At present BChE is the gold standard for assaying exposure to nerve agents and organophosphorus pesticides.

**Response:** The statement could be true that Bche was primarily studied for toxicity caused by BChE may be an old statement, however our method has been worked on AchE and an in-silico study by Chowdary S et al., 2018 has reported that the interactions observed in the most favorable Total Interaction Energy pose of ACh and 1-NA supporting our study that the 1-Naphthol acetate would be a most favourable to Ache over Bche. As per the comment from the reviewer we have deleted the previous statement modified the statement with the above response.

**Comment 13:** “The steric hinderance due to the butyl structure in the Bche also interferes and prevents the formation on 1-Naphthol from 1 Naphthol Acetate which clearly explains the effect of Bche activity in blood. In case of butyl acetylene cholinesterase, the spatial arrangement of atoms is sterically crowded.” The authors have misunderstood the literature on the crystal structures of AChE and BChE. AChE has a very narrow channel for accommodating substrates and inhibitors. In contrast BChE has a wide-open area leading to the active site gorge and a large space at the bottom of the active site gorge. Therefore, BChE can interact with large molecules, but AChE is restricted to interacting with small molecules.

**Response:** We apologise for our comment made previously and agree with the reviewer comment on the active sites, we have deleted the statement on steric hinderance effect on the substrates. We are thankful to the reviewer for the valuable comment and understanding of the interaction pockets for us.

**Comment 14:** Please check the author names in reference 36 and reference 40

**Response:** We thank the reviewer for the comment, we have corrected the errors thet were found in the references.

**Comment 15:** The name of author Simas in reference 40 is written Simas, B.C.A. but in reference 41 is written Simas, A.B.C.

**Response:** We have cross checked with the citations again and the same has been corrected in the revised manuscript. The author in reference 40 was cited as Simas, B.C.A while in reference 41 it was represented as Simas, A.B.C. as extracted from the published citations.
