# Supplementary material for: A novel RP-HPLC method for quantification of cholinesterase activity in human blood: An application for assessing organophosphate and carbamate insecticide exposure
Source: PLoS One. 2022 Dec 30;17(12):e0279287. doi: 10.1371/journal.pone.0279287 (PMC9803206; doi:10.1371/journal.pone.0279287)
Supplement: Attachment — Submitted filename: Respone to reviewer comments final.docx [file pone.0279287.s003.docx]

**Response to Reviewer**

**Reviewer #1:**

The reviewer’s comments are in **blue color** and the author’s responses are in **black colo**r. The author incorporated some changes in the revised manuscript and highlighted them in red color with track changes.

Thank you very much for your valuable suggestions, and comments, and for considering this manuscript. The author is grateful for the suggestions and recommendations made by you to improve the quality of our manuscript. The manuscript is now revised carefully according to the Editor’s and best reviewer’s suggestions and included it in the detailed point-to-point answers mentioned below.

**Comment 1. Page 5 line 109. Please change 1 Mole to 1 M.**

**Response to Reviewer #1** **comment 1:** We thank the reviewer for the correction. According to your suggestion, We corrected the spelling. and we have corrected the error and included it in the revised manuscript (line number 110, page no:6).

**Comment 2. Page 9 line 190 states that the assay was stopped by addition of 500 µl acetonitrile. However, line 204 and the scheme show the assay was stopped with 700 µl acetonitrile. Please clarify this inconsistency.**

**Response to Reviewer #1 comment 2:** Thank you for your valuable finding and recommendations made by you to improve the quality of our manuscript. We apologize for the inconsistency in the data representation and typographical representation. we have corrected and rewritten and included it in the revised manuscript (Line number 192-194; page number 9)

**Comment 3. Page 9 lines 187-192. I do not understand why you think addition of 50 µl water to 50 µl naphthyl acetate and 400 µl AChE in phosphate buffer is a test of the role of AChE in ester hydrolysis. This section would make sense if the assay included no AChE, so that you would be testing the spontaneous hydrolysis of the ester.**

**Response to Reviewer #1 comment 3:** We thank and are grateful to the reviewer for the valuable comment because he highlighted the genuine suggestion to us, to improve the quality of our manuscript. The peak of 1 naphthol was obtained in the presence of AChE , while in absence of AChE in similar condition (in water) no peak of 1 naphthol was obtained. This experiment was performed to check autohydrolysis of 1-naphthol acetate in the presence of water. We have corrected and incorporated it in the revised manuscript as per your suggestion (line number 194-196 page number 9)

**Comment 4. Page 9 line 186. If you are testing spontaneous hydrolysis of naphthyl acetate, then the title of this section should be changed to “Auto hydrolysis of napththyl acetate in the absence of AChE”.**

**Response to Reviewer #1 comment 4:**Thank you for your comment according to your suggestion we have changed the title in the revised manuscript line number 187 page no : 9

**Comment 5. Page 10 line 212. Please delete “in 2.6.1”**

**Response to Reviewer #1 comment 5:**Thank you for your comment according to your suggestion we have corrected it in the revised manuscript line number 218 page no : 10

**Comment 6. Page 11 line 230. Please define a unit of activity.**

**Response to Reviewer #1 comment 6:**Thank you for your comment according to your suggestion we have incorporated the definition of the unit of activity “The enzyme activity is measured in units which indicate the rate of the reaction catalyzed by that enzyme expressed as micromoles of substrate transformed (or product formed) per minute” in the revised manuscript line number 236-238 page no : 11

**Comment 7. Page 14 line 351. The statement is incorrect that the Ellman method utilizes 10 µl of plasma sample. The original Ellman method used whole blood. The Ellman method has been adapted to assay BChE activity in plasma samples, but plasma activity was not measured by Ellman in their classic 1961 paper.**

**Response to Reviewer #1 comment 7:**Thank you for your comment, instead of plasma we have added whole blood and incorporated it in the revised manuscript the line number 354 page number 18

**Comment 8. Page 20 line 396. The statement that your HPLC assay is the best analytical approach is not convincing. You have no experience with other assays, but rely on literature reports to make your claim.**

**Response to Reviewer #1 comment 8:** Instead of best we changed to “suitable” in line number 393 page number 20

**Comment 9. Page 20 line 408. “synthetic AChE” do you mean recombinant AChE?**

**Response to Reviewer #1 comment 9:** Yes I agree, it is Recombinant AChE and incorporated it in line number 398 and page number 20

**Comment 10. The Discussion should be shortened.**

**Response to Reviewer #1 comment 10:** We have rewritten, shorten the discussion, and removed the repeated sentences in the revised manuscript.
